# Simple spectrophotometric assay for measuring catalase activity in biological tissues

**DOI:** 10.1186/s12858-018-0097-5

**Published:** 2018-08-03

**Authors:** Mahmoud Hussein Hadwan

**Affiliations:** Chemistry Department College of Science, University of Babylon, Hilla City, Babylon Governorate P.O. 51002 Iraq

**Keywords:** Catalase activity, Hydrogen peroxide, Carbonato-cobaltate (III) complex, Cobalt, Bicarbonate

## Abstract

**Background:**

The details of a precise, accurate, and sensitive spectrophotometric method for measuring catalase activity are presented here. The assay was established for biological samples and depends on the rapid formation of a stable and colored carbonato-cobaltate (III) complex. Samples exhibiting catalase activity are incubated with hydrogen peroxide solution for 2 min prior to rapid mixing of the incubation enzymatic reaction mixture with cobalt-bicarbonate reagent, which assesses non-reacting hydrogen peroxide. Catalase activity is always directly proportional to the rate of dissociation of hydrogen peroxide. Hydrogen peroxide acts to oxidize cobalt (II) to cobalt (III) in the presence of bicarbonate ions; this process ends with the production of a carbonato-cobaltate (III) complex ([Co (CO_3_)_3_]Co). The formed end product has two maximum absorbance peaks: 440 nm and 640 nm. The 440-nm peak has been utilized for assessing catalase activity.

**Results:**

The catalase activity results of the current method for erythrocyte lysate homogenates were computationally identical to those of the dichromate method (*r* = 0.9950). The coefficient of variation was calculated to determine the imprecision of the current assay. The within-run and between-run results were 2.96 and 3.83%, respectively.

**Conclusion:**

This method is appropriate for analyzing bacteria, red blood cells and liver and kidney tissue homogenates.

## Background

Catalase (EC 1.11.1.6) is an important enzyme that acts to dissociate hydrogen peroxide (H_2_O_2_) into molecular oxygen (O_2_) and water (H_2_O) [1]. Catalase has a molecular weight equal to 250 kDa and consists of four hemoprotein groups [2]. Like other antioxidant enzymes, catalase is also present in plants and animal cells such erythrocytes, renal cells and hepatic cells [1, 3]. Catalase is also produced by a wide spectrum of prokaryotic and eukaryotic organisms. It is an intracellular enzyme that has been discovered in most facultative anaerobes and all aerobic bacteria, but is not present in obligate anaerobes [4]. Catalase is the second most abundant enzymatic antioxidant (after superoxide dismutase), which attenuates the levels of reactive oxygen species that ubiquitously accompany pathological disorders such as aging, cataract, cancer, nutritional deficiency, atherosclerosis, and diabetes [5].

There has been substantial progress in the development of efficient methods for assessing catalase activity in the fields of clinical pathology, biotechnology, and applied microbiology because of the spread of microbial populations in a variety of foods [6–17]. Several of these methods have been reported.

The principally common method for measuring catalase activity is the UV spectrophotometric method, which depends on monitoring the change of 240 nm absorbance at high levels of hydrogen peroxide solution (≥30 mM). High levels of hydrogen peroxide (H_2_O_2_) immediately lead to inhibition of the catalase enzyme by altering its active site structure, although there is variation in the extent to which this occurs. Additionally, there is a need for a method for continuously assessing low catalase activity against a high background level of absorbance because many cellular constituents, such as nucleic acids and proteins, exhibit intense absorption at 240 nm [18].

Other methods of measuring catalase activity have been developed, including those involving iodometry [19] chemiluminescence [14], polarimetry [15], and monitoring the production of oxygen via an oxygen electrode [20] or a low-flow gas meter [21]. These methods are time-consuming and inappropriate for clinical application. Alternatively, catalase activity can be measured quantitatively by titration of the unreacted excess of hydrogen peroxide [22]. However, this method may be associated with difficulty in end-point determination [23].

This paper reports a simple assay for measuring catalase activity that includes the measurement of hydrogen peroxide spectrophotometrically. This method is free from the interference that results from the presence of amino acids, proteins, sugars, and fats in the studied sample.

## Results

**Fig. 1:**
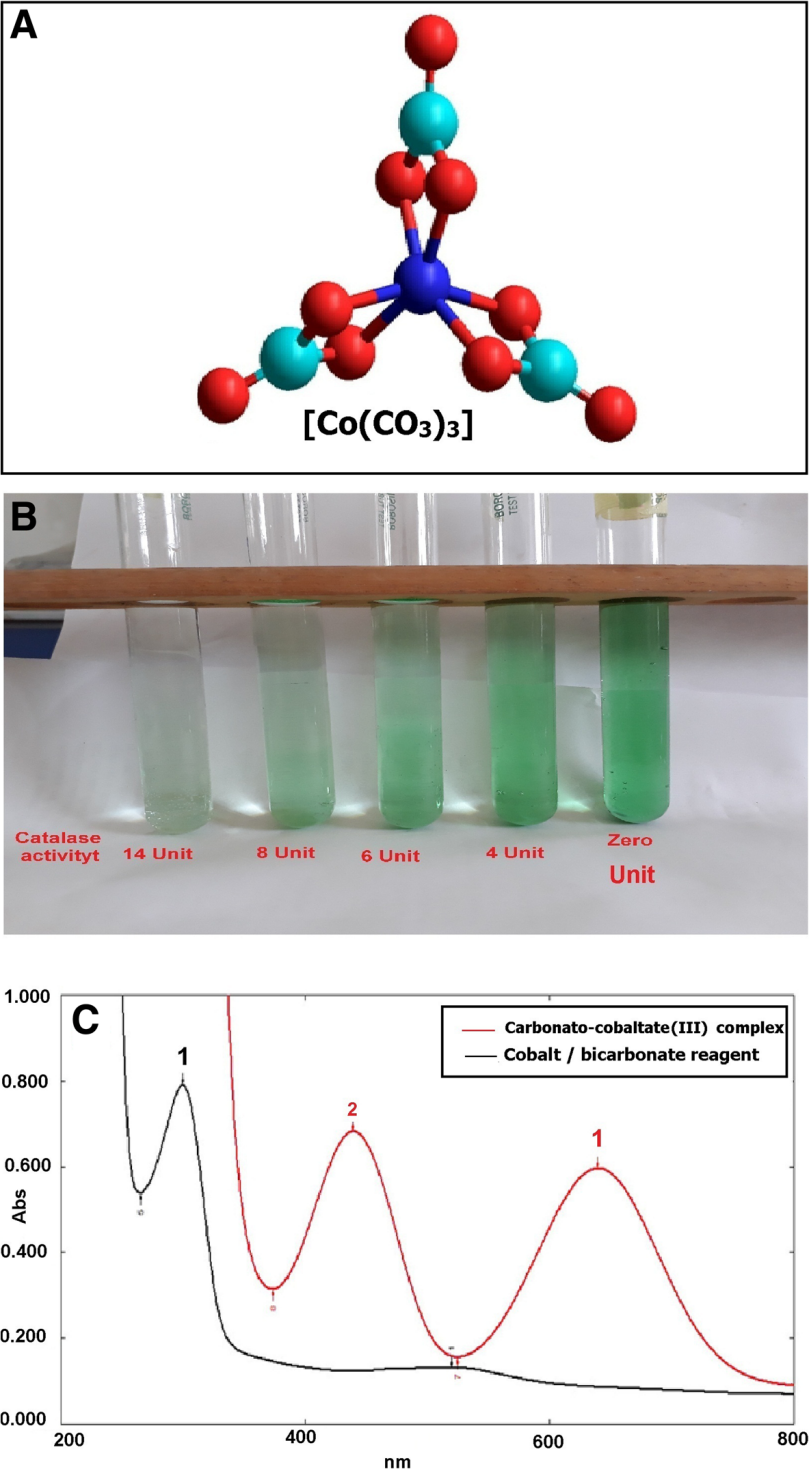
The spectrophotometric properties of the carbonato-cobaltate(III) complex that correlates with the activity of catalase enzyme. **a** A carbonato-cobaltate(III) complex. **b** Decreased color intensity in association with increased catalase activity. **c** Absorption spectra obtained for the colorimetric products of the present assay

Cobalt-bicarbonate solution can act as a” stop bath” for reactions regulated by the catalase enzyme. Immediately after mixing the cobalt-bicarbonate reagent with the enzyme reaction solution, its content of cobalt (II) is oxidized to cobalt (III); any unreacted hydrogen peroxide resulting from the catalase activity will oxidize the cobalt (II) to cobalt (III) and then react with carbonate to produce a carbonato-cobaltate (III) complex ([Co (CO_3_)_3_]Co) (Fig. 1a), which has an intense olive green color. Catalase activity is always directly proportional to the rate of dissociation of hydrogen peroxide in the used samples. The decrease of color intensity can be used as an index to represent the increase catalase activity (Fig. 1b). The spectrum of the colored end product [carbonato-cobaltate (III) complex ([Co (CO_3_)_3_]Co)] was scanned from 200 to 700 nm and showed bands at 440 and 640 nm. The 440-nm band was used for measuring catalase activity (Fig. 1c).

To examine the potential impacts of chemical interference that might change the catalase activity, nine chemicals suggested to interfere with this activity were prepared by mixing 1 ml of catalase with known activity (5 U/ml) and 9 ml of a specific interfering substance dissolved in phosphate buffer (50 mM, pH 7.4). The resulting catalase activity was calibrated using the dichromate method as explained by Sinha [7] and modified by Hadwan [8]. The final activity equaled 0.5 U.mL^− 1^. Table 1 shows the effects of various types of interference on the catalase assay.

**Table 1:** The effects of several interfering chemicals on the activity of the catalase enzyme

Supposed chemical interferences	Concentration of supposed chemical interferences	Added catalase U mL^− 1^	Found catalase U mL^− 1^	Relative error (%)
Heparin	78.4 USP/10 mL	0.5	0.509	−1.8
EDTA	20.0 μM	0.5	0.511	−2.2
Glucose	0.35 mg mL^−1^	0.5	0.509	−1.8
Histidine	50 μM	0.5	0.492	1.6
Albumin	0.5 mg mL^−1^	0.5	0.516	−3.2
Ascorbic acid	20.0 μM	0.5	0.489	2.2
Arginine	50 μM	0.5	0.507	−1.4
Uric Acid	50 μM	0.5	0.495	1
Lysine	50 μM	0.5	0.509	−1.8

The results obtained for each sample by the present assay were compared with those obtained by the dichromate method as described previously by Sinha [7] and modified by Hadwan [8]. Identical buffer, substrate, and sample were used in both procedures. The results of the current method showed good precision (Table 2) and good correlation with the dichromate-dependent assay (Table 3).

**Table 2:** The precision of the present method

	No.	Mean (± SD): U mL^− 1^	CV %
Within-run	20	6.4 ± 0.19	2.96%
Between-run	20	6.6 ± 0.26	3.83%

*SD* standard deviation

**Table 3:** Statistical examination of the results achieved for catalase activity by the dichromate and current methods (U mL^− 1^)

No. of experiments	20
Mean of dichromate assay	6.61
Mean of the present assay	6.45
Mean of both assays	6.53
Regression coefficient B	0.9972
Regression coefficient A	0.0028
Correlation coefficient	0.995

The accuracy of the current method was calculated by the recovery of activity of the catalase enzyme that had been added to the reaction solution. The catalase enzyme was purchased from HiMedia Laboratories (New Delhi, India, product code: TC037). It was prepared in 0.05 mM phosphate buffer solution and calibrated using the dichromate method, as documented previously by Sinha [7] and modified by Hadwan [8]. The results are detailed in Table 4.

**Table 4:** Analytical recovery of activity of catalase enzyme added to the reaction solution

Catalase enzyme contents	Catalase enzyme activity added U mL^−1^	Catalase enzyme calculated activity U mL^− 1^	Catalase enzyme observed activity^a^ U mL^− 1^	Recovery %
Enzymatic sample	–	–	5	–
Enzymatic sample + catalase enzyme added	1	6	5.85	97.5%
Enzymatic sample + catalase enzyme added	3	8	7.9	98.75%
Enzymatic sample + catalase enzyme added	5	10	9.82	98.2%
Enzymatic sample + catalase enzyme added	7	12	12.11	100.9%
Enzymatic sample + catalase enzyme added	8	13	12.75	98.2%
Enzymatic sample + catalase enzyme added	9	14	12.7	90.8%
Enzymatic sample + catalase enzyme added	10	15	12.75	85%

^a^mean of triplicate determinations

**Fig. 2:**
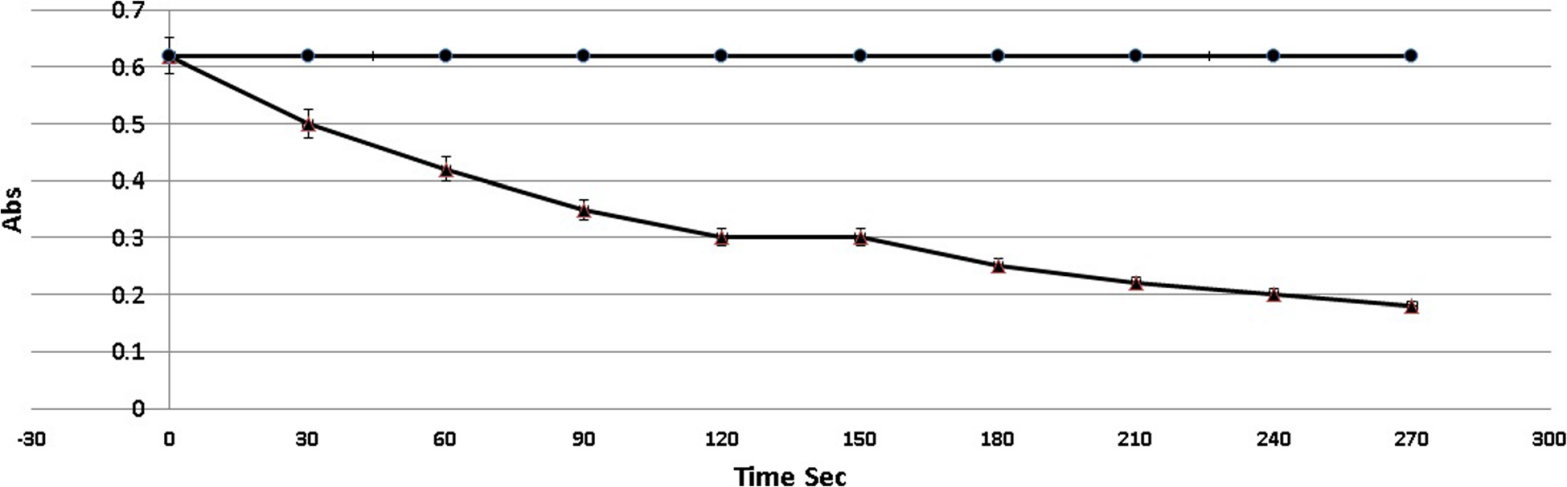
The absorbance of carbonato-cobaltate(III) complex ([Co(CO_3_)_3_]Co) as a function of the incubation time of H_2_O_2_ alone (●) or H_2_O_2_ mixed with catalase enzyme (0.86 U mL−1) (▲)

Conversion of hydrogen peroxide (H_2_O_2_) to molecular oxygen and water (H_2_O) in a chemical reaction regulated by catalase was monitored by determining the absorbance of the carbonato-cobaltate (III) complex as a function of time (as shown in Fig. 2). An ideal reaction time of 120 s was selected in the present method because this corresponded to the time when the absorbance plateau was reached, reflecting optimal hydrogen peroxide dissociation by the activity of the catalase enzyme.

**Fig. 3:**
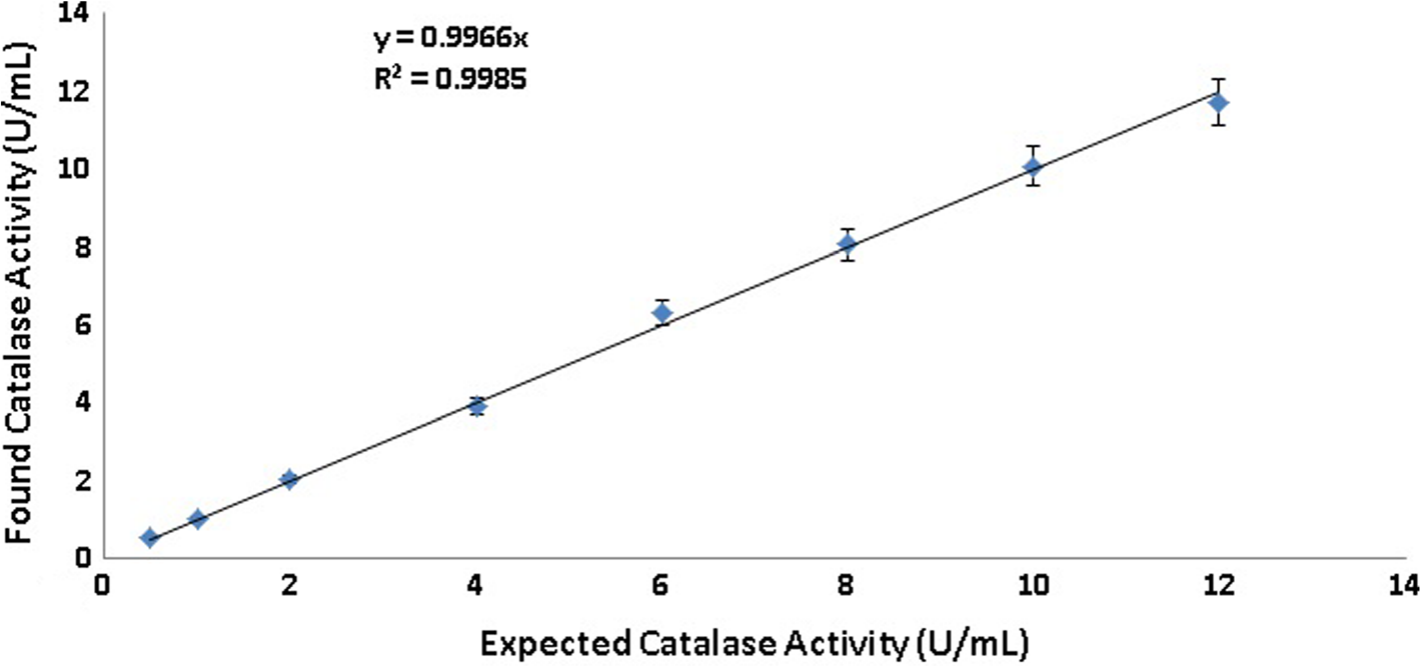
The catalase activities measured by the carbonato-cobaltate(III) complex method versus those expected for red blood cell homogenate at different dilutions

The dilution sensitivity for red blood cell homogenate was evaluated using a carbonato-cobaltate (III) complex method. The identified catalase activities (instrumental measured activity) were determined and plotted against the expected catalase activity at a series of dilutions of red blood cell homogenate (Fig. 3). Figure 3 reveals the good correlation between these variables (*r* = 0.9985).

**Fig. 4:**
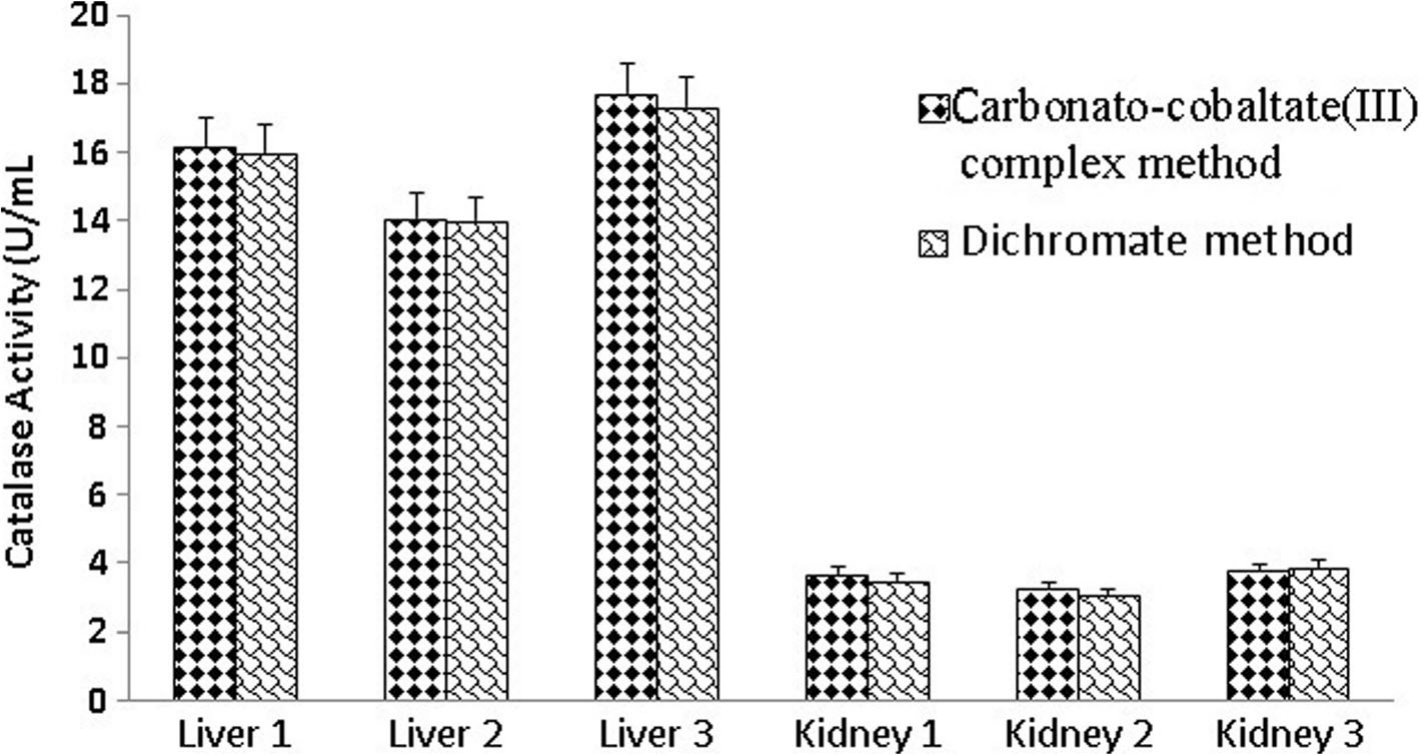
The catalase activity of liver and kidney tissues homogenates measured with the carbonato-cobaltate(III) complex method in comparison to that with the dichromate method

The carbonato-cobaltate (III) complex method was utilized to measure the catalase activity of liver and kidney tissue homogenates (1:500 diluted homogenate). These tissues were normally shown to exhibit higher catalase activity than other tissues (Fig. 4).

**Fig. 5:**
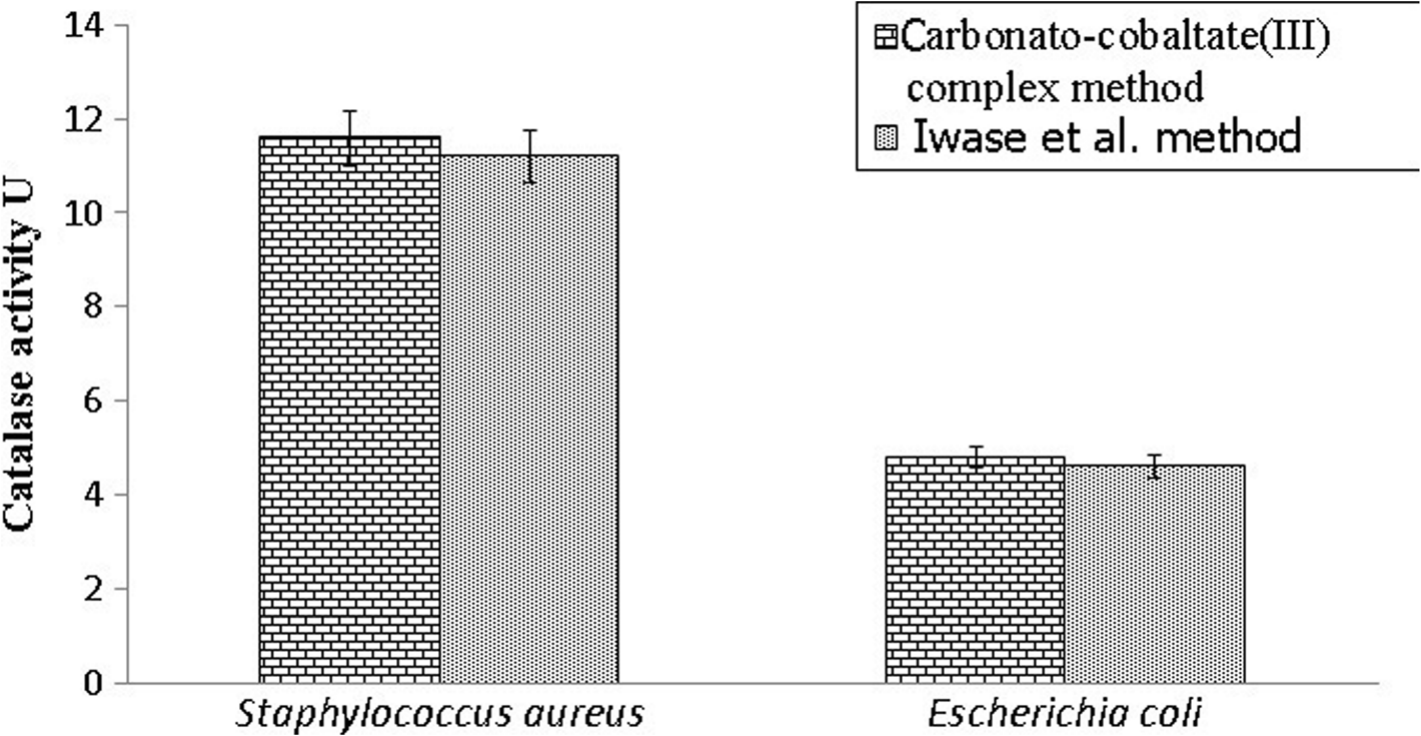
The catalase activity of *Escherichia coli* and *Staphylococcus aureus* measured with the carbonato-cobaltate(III) complex method in comparison to that with the Iwase et al. [26]

The present method was applied to measure the catalase activity in bacteria. Two bacterial laboratory strains were used for this (*Escherichia coli* and *Staphylococcus aureus* were isolated and diagnosed by Hussein O.M. Al-Dahmoshi/Advanced Microbiology Lab, College of Science, University of Babylon, Iraq). *Staphylococcus aureus* was normally shown to exhibit higher catalase activity than *Escherichia coli* (Fig. 5).

## Discussion

This paper describes a new method for assessing catalase activity in various biological samples. This method depends on the oxidation of cobalt (II) to cobalt (III) by hydrogen peroxide in the presence of bicarbonate solution to produce a carbonato-cobaltate (III) complex ([Co (CO_3_)_3_]Co). The reaction was shown to be very stable in terms of the end product produced for samples of hydrogen peroxide, with no significant change in the absorbance at 440 nm being observed at room temperature until 5 h. This result agrees with the results obtained by Masschelein et al. [24].

According to the results shown in Table 1, the presence of vitamins, amino acids, proteins, and antioxidants in biological fluids did not interfere with the currently proposed method of assessing catalase activity. The absence of interference in the current method is due to its dependence on the basis of the oxidation property of the hydrogen peroxide on cobalt (II). When we consider that the concentration of oxidants in biological samples is very low compared with the hydrogen peroxide concentration used in catalase assessment methods (5–50 mM), this should explain the absence of interference.

The results of the high precision and accuracy of the current method as indicated in Tables 2 and 3 reveal the suitability of this method for clinical and research applications. The present assay is effective for measuring low concentrations of hydrogen peroxide (nontoxic and physiological concentrations), to which the frequently used UV-dependent method is not precisely responsive. As a result, the usual difficulties of the UV-dependent method, including comparatively low selectivity and sensitivity, and disturbance of absorbance due to the evolution of gaseous oxygen, can be overcome.

The results summarized in Table 4 show that the linearity of the following method reaches about 12.75 U mL^− 1^. The limit of quantification (LOQ) and limit of detection (LOD) of the method based on cobalt/bicarbonate reagent for catalase assessment were found to be 0.04 and 0.012 U mL^− 1^, respectively.

Based on these results, the LOD and the linearity for the current method were better than those of other previously described methods [7–14], while the precision (within-run and between-run precision) and accuracy were identical. In terms of the methodology, this assay is preferable to estimate catalase activity compared with other biochemical methods.

The present method was utilized to measure catalase activity in liver and kidney tissue homogenates. The expected results were obtained, i.e., liver and kidney homogenates were generally shown to be associated with higher catalase activity than other tissues (Fig. 4), which may be due to the role of these organs in the detoxification of reactive oxygen species [25]. The catalase activity levels measured with the carbonato-cobaltate (III) complex method were compared with those obtained by the dichromate method as described previously by Sinha [7] and modified by Hadwan [8] (Fig. 4). The level of catalase activity of tissue homogenates measured with the new carbonato-cobaltate (III) complex method was found to be compatible to that measured by the dichromate method.

In addition, the developed method could measure the catalase activity in bacteria. Two laboratory strains were used in the current study (*Escherichia coli* and *Staphylococcus aureus*). The levels of catalase activity of the studied bacteria obtained by using the proposed carbonato-cobaltate (III) complex method were found to be compatible with those obtained by the method reported previously by Iwase et al. [25]. *Staphylococcus aureus* was normally shown to exhibit higher catalase activity than *Escherichia coli* (Fig. 5).

## Conclusions

This paper describes a simple method for assessing the activity of the catalase enzyme, which can be completed with only a few steps. This method allows the measurement of catalase enzyme activity in biological samples that contain high concentrations of vitamins, amino acids, proteins, antioxidants, or other interfering substances, as well as at low concentrations of hydrogen peroxide. The cobalt/bicarbonate solution is a sensitive reagent for hydrogen peroxide, which facilitates the assessment of catalase enzyme at low concentrations of substrate, confirming that the auto-inactivation of catalase is reduced during the assessment steps.

## Methods

### Principle

The current method is based on the concept of establishing a simple assay of catalase enzyme activity for biological tissues, which depends on the conversion of the oxidation state of cobalt (II) to cobalt (III) by hydrogen peroxide in the presence of bicarbonate solution. This process ends with the formation of a carbonato-cobaltate (III) complex ([Co (CO_3_)_3_]Co). This end product has two clear absorption peaks at 440 and 640 nm. The 440-nm band has been used for the assessment of catalase activity. Dissociation of hydrogen peroxide is proportional to the activity of catalase enzyme in the used sample. The method has been developed for the measurement of catalase activity in biological samples (bacteria, red blood cells, and liver and kidney tissue homogenates).

### Chemicals

The catalase enzyme was purchased from HiMedia Laboratories (New Delhi, India, product code: TC037). All other chemicals were provided from standard commercial suppliers and were of analytical grade.

### Reagents

1. Cobalt (II) solution was prepared by dissolving 20.3 g of Co (NO_3_)_2_·6H_2_O in 1 l of distilled water.
2. Sodium hexametaphosphate solution (Graham salt) was prepared by dissolving 10 g of (NaPO_3_)_6_ in 1 l of distilled water.
3. Sodium bicarbonate solution was prepared by dissolving 180 g in 2 l of double distilled water.
4. Working solution: This consisted of 100 ml of cobalt (II) solution, 100 ml of Graham salt solution, and 1800 ml of sodium bicarbonate solution, which were mixed well after preparation (the order in which these substances are added is very important for obtaining accurate results).
5. Phosphate buffer (pH 7.0, 50 mM) was prepared by mixing the following solutions (a & b) at a ratio of 1:1.5 [(a) 6.81 g of KH_2_PO_4_ was dissolved in 1 l of double distilled water and (b) 8.90 g of Na_2_HPO_4_.2H_2_O was dissolved in 1 l of double distilled water].
6. Hydrogen peroxide (10 mM) was prepared by adding 0.1134 ml of 30% hydrogen peroxide to 100 ml of phosphate buffer; this solution was freshly prepared and calibrated daily depending on the molar extinction coefficient of hydrogen peroxide, which equals 43.6 M^− 1^ cm^− 1^ at 240 nm.
7. Erythrocyte lysate preparation: Three milliliters of whole blood was drawn from an anonymous donor by peripheral venous puncture. The whole blood was transferred into heparinized tubes to prevent coagulation. The centrifugation process was performed after 10 min at 400×g for 10 min, followed by separation and disposal of the plasma and buffy coat. The next step included washing 500 μl of the remaining red blood cell sediment three times with 5 ml of 0.9% NaCl solution, with centrifugation at 400×g for 10 min after each wash. Subsequently, 2 ml of ice-cold double distilled water was transferred into a test tube containing 500 μl of erythrocyte sediment (fivefold dilution), vortexed for 5 s, and incubated for 15 min at 4 °C in the dark. The final step included dilution of the resulting 2.5 ml of fivefold re-suspended stock hemolysate with phosphate buffer solution (0.05 M) to reach a dilution factor of 500. The resulting hemolysate solution was used as a source for catalase activity.
8. Tissue preparation: Male albino rats (100 g) were originally purchased from the animal house of the College of Science, University of Babylon, Iraq. Animals were housed in polypropylene cages under hygienic and standard environmental conditions (28 °C ± 2 °C, humidity 60–70%, 12 h light/dark cycle). The animals were allowed a diet and water ad libitum. Just before the tissue catalase activity measurements, the rats were anesthetized with IP sodium pentobarbital (75 mg/kg) and were sacrificed by open pneumothorax, and their kidney and liver tissues were surgically excised. Immediately after the surgery, a solution consisting of 0.9% (*w*/*v*) NaCl was used to wash the tissue samples; this step eliminated external contaminants (blood, etc.). The next step included weighing and homogenizing the tissues using a cold 1.15% (w/v) potassium chloride solution in a glass homogenizer. The homogenate solution was filtered and diluted (at a ratio of 1:500) with 0.05 M phosphate buffer solution before analysis. The homogenates were immediately used for the assessment of catalase activity.

All clinical experiments that include human blood samples or animal tissues were approved by the Ethics Committee of the University of Babylon/College of Science (approval number A7/2017). All procedures and protocols were performed according to the Declaration of Helsinki. The signed written consent of the participant (41 years old) included in the study was obtained. Informed consent was obtained before beginning the study.

#### Instrument:

A spectrophotometer (Shimadzu 1800) was used in the current study.

#### Procedure:

As shown in Table 5.

**Table 5:** The steps of the procedure used for assessing the activity of catalase

Reagents	Test	Standard	Blank
Catalase source sample	500 μl	–	–
Distilled water	–	500 μl	1500 μl
Hydrogen peroxide	1000 μl	1000 μl	–
The tubes were mixed with a vortex and incubated at 37 °C for 2 min, after which the following substance was added:			
Working solution	6000 μl	6000 μl	6000 μl
Next, the tubes were vortexed for 5 s and then kept at room temperature for 10 min in the dark. The changes in absorbance were recorded at 440 nm against the reagent blank			

#### Calculation:

The rate constant of a first-order reaction (k) equation was used to determine catalase activity:

1 $$ \mathrm{Catalase}\ \mathrm{Activity}\ \mathrm{of}\ \mathrm{test}\ \mathrm{kU}={\frac{2.303}{\mathrm{t}}}^{\ast}\mathrm{l} og\frac{{\mathrm{S}}^{{}^{\circ}}}{\mathrm{S}} $$

t: time.

S°: absorbance of standard tube.

S: absorbance of test tube.

## Abbreviations

(H_2_O_2_): hydrogen peroxide
(NaPO_3_)_6_: Sodium hexametaphosphate
Cat: catalase
Co (NO_3_)_2_·6H_2_O: Cobalt (*II*) nitrate hexahydrate
